# Pthisis Pulmonalis in New Orleans

**Published:** 1845-09-01

**Authors:** 


*Pthisis Pulmonalis in New Orleans.*—The New Orleans Medical Journal, (which by the way, is one of the most spirited and ably conducted journals of our country,) for July ult., contains a list of the deaths and diseases in the city of New-Orleans for the months of April and May last. The total number of deaths during these months was 502, of this number 451 were adults. We are surprised to observe that among the diseases, cases of Pthisis bear by far the greater proportion, being in both months 73. In 51 cases the diseases were unknown. In the remainder the diseases are specified with particularity and exactness. The report purports to emanate from Dr. Lewis, Secretary to the Board of Health, and exhibits internal evidence of precision and care. It is greatly to be regretted, that we have not in our city effectual provisions for a correct registration of deaths and diseases. It has been once or twice attempted, but the measures have been inadequate to the object, and it appears now to be entirely abandoned. The true and only method to ensure authentic bills of mortality, is to commit the business to a competent medical man, invested with proper official power and responsibility. The duty might be connected with the present office of City Physician. If it were made necessary for Undertakers or Sextons to report all deaths to the City Physician, he being bound to ascertain by personal inquiry of the attending physician the disease in all cases, it seems to us that the matter would be provided for. It is certainly a desirable and important end, and we beg leave to commend it to the attention of our city authorities.

